# Unraveling the transcriptomic landscape of eye migration and visual adaptations during flatfish metamorphosis

**DOI:** 10.1038/s42003-024-05951-x

**Published:** 2024-03-01

**Authors:** Laura Guerrero-Peña, Paula Suarez-Bregua, Lucía Sánchez-Ruiloba, Luis Méndez-Martínez, Pablo García-Fernández, Ricardo Tur, Juan J. Tena, Josep Rotllant

**Affiliations:** 1 Aquatic Biotechnology Lab., Institute of Marine Research, Spanish National Research Council (IIM-CSIC), 36208 Vigo, Spain; 2 Institute of Marine Research, Spanish National Research Council (IIM-CSIC), 36208 Vigo, Spain; 3 Nueva Pescanova Biomarine Center, S.L., 36980 O Grove, Spain; 4 Centro Andaluz de Biología del Desarrollo (CABD), CSIC-Universidad Pablo de Olavide, 41013 Sevilla, Spain

**Keywords:** Biological metamorphosis, Gene regulatory networks

## Abstract

Flatfish undergo a remarkable metamorphosis from symmetrical pelagic larvae to fully asymmetrical benthic juveniles. The most distinctive features of this transformation is the migration of one eye. The molecular role of thyroid hormone in the metamorphosis process in flatfishes is well established. However, the regulatory network that facilitates eye movement remains enigmatic. This paper presents a morphological investigation of the metamorphic process in turbot eyes, using advanced imaging techniques and a global view of gene expression. The study covers migrant and non-migrant eyes and aims to identify the genes that are active during ocular migration. Our transcriptomic analysis shows a significant up-regulation of immune-related genes. The analysis of eye-specific genes reveals distinct patterns during the metamorphic process. Myosin is highlighted in the non-migrant eye, while ependymin is highlighted in the migrant eye, possibly involved in optic nerve regeneration. Furthermore, a potential association between the *alx3* gene and cranial restructuring has been identified. Additionally, it confirmed simultaneous adaptation to low light in both eyes, as described by changes in opsins expression during the metamorphic process. The study also revealed that ocular migration activates systems asynchronously in both eyes, providing insight into multifaceted reorganization processes during metamorphosis of flatfish.

## Abstract (web summary)

Flatfish eye migration in metamorphosis study shows up-regulated immune genes. The process involves roles of myosin/ependymin, the link of *alx3*’s to cranial restructuring, changes in opsins adaptation to low light and an asynchronous system activation.

## Introduction

The visual system is particularly important in predatory fish for their feeding and hunting behavior. This has forced animals, such as flatfish, to adapt their visual system to environmental conditions. Flatfish, an important group of fish with a high economic value, undergoes one of the most epic metamorphoses in the animal kingdom involving morphological and behavioral changes: a symmetric pelagic larva becomes an asymmetric benthic juvenile, including the migration of one eye to the other side of the body. How these fish have adapted to their new life conditions has been an enigma that has concerned scientists from Darwin to the present day^1^. It is believed that this unusual morphology and eye migration in flatfish has evolved as a response to the need for ambushing or seeking refuge^2,3^.

The transition from pelagic to benthic life requires adaptation to environmental photic variations. Flatfish undergo a complex reorganization of the retina’s photoreceptors during metamorphosis to adapt to a demersal lifestyle^4–6^. As both eyes experience attenuation of light conditions, no differences related to development are found in the ocular layers or lens^7^. However, several asymmetric characteristics related to eye position and visual system have been described in the literature. In order to accommodate the eye on the ocular side, a different orbit composition has been described in migrant and non-migrant eyes, such as a shorter extraocular muscle in the migrant eye^8^. Furthermore, there is an asymmetric craniofacial remodeling during lateralization, excluding the jaws, which remain mainly symmetrical^9–11^. Regarding differential features between eyes directly related to the visual system, a clear asymmetry in the length of the optic nerve has been found, with the non-migrant eye having a shorter nerve^12^, as well as a tendency for the optic nerve of the migrant eye to cross over the non-migrant eye at the optic chiasm^13^. Brinon et al.^14^ suggested a lower functionality of the migrant eye, such as a smaller left optic tectum size during metamorphosis. However, there appear to be no differences in hunting success during settlement.

Understanding the mechanisms driving eye migration has been a challenge that is still far from being fully unraveled. It is well-studied that thyroid hormone, as in amphibians^15^, is responsible for triggering metamorphosis and, therefore, eye migration^16^. Thyroid hormone inhibitors result in incomplete migration and symmetrical juveniles^17^. Different hypotheses have tried to explain eye migration in flatfish: (i) cranial asymmetry drives eye movement^18^; (ii) cell proliferation in the suborbital tissue on the blind side pushes the eye to the other side of the body^19^. Despite these great contributions, there are not enough studies that shed light on what occurs in the eye at a molecular level. We propose a transcriptome-wide study of the left and right eye, including surrounding tissue, at three key developmental stages (pre-metamorphic, climax of metamorphosis, and post-metamorphic) in turbot (*Scophthalmus maximus)* with the aim of revealing the differentiation of gene expression between an eye undergoing migration (right eye in turbot) and the eye remaining in its position after settlement (left eye in turbot).

## Methods

### LSFM rapid 3D imaging of skull bone development during turbot metamorphosis

A total of 50 fish reared under standard production conditions at 18 °C, were collected following a temporal sequence (10 individuals per stage): 10, 15, 20, 30, 57 dpf (days post fertilization). Turbot fish were euthanized using MS-222 (500 mg/L, for 20–30 min) (Sigma-Aldrich, USA), followed by fixation in 2% paraformaldehyde. To facilitate bone observation, fish were stained with Alizarin Red protocol specifically modified for turbot larvae and juvenile^20^. The stained animals were then preserved and observed in glycerol. To observe in detail the bone structure of the pre-selected turbot stages, samples were analyzed using a Zeiss Lightsheet7 imaging system (LSFM). The images were captured using a 5× NA 0.16 plan-neofluid detection objective and a zoom of 0.5 to achieve a larger field of view. The illumination laser was set to 561 nm, combined with an SBS LP 640 detection filter to capture the alizarin red signal. The Pivot Scan system was employed to eliminate any shadowed areas that might appear in the field. The images were acquired at a resolution of 1.88 × 1.88 × 4.73 mm. For each specimen, a suitable number of tiles were selected to cover the entire sample at lateral position (between 1 × 1 and 4 × 4). In the *z*-axis, all planes with signals were recorded. After the acquisition, tile stitching was performed using the Zen Blue software from Zeiss. Due to the large size of the samples, images were taken from two angles (0 and 180°), which were then fused using the VolumeFusion tool in the Arivis 4D software (Zeiss, Germany). Subsequently, 3D visualizations of each specimen were created using the Arivis 4D software. Structures of interest, such as the skull, bone, pseudomesial bar, and mandible, were selected using the wand tool to enhance the asymmetry of the individuals throughout the development stages. Finally, different 3D views (lateral, dorsal, and frontal) were generated for each individual.

### RNAseq sample collection

Three key stages in turbot development were defined based on Al-Maghazachi and Gilson^21^ and Suarez-Bregua’s^22^ morphological criteria. Fish reared under standard production conditions at 18 °C, were collected from three metamorphic stages: pre-metamorphic (15 dpf, symmetrical larvae); the climax of metamorphosis (30 dpf, the migratory eye is visible from the other side of the body); post-metamorphic stage (57 dpf, both eyes lodged in the ocular side). Three independent replicates per stage were collected, and fish were euthanized with a lethal dose of MS-222 (500 mg/L for 20–30 min) (Sigma-Aldrich, USA).

### RNA isolation and sequencing

The migrant and non-migrant eyes and their respective surrounding tissue were extracted separately for each stage. In order to obtain enough RNA quantity for sequencing, three eyes per replicate were pooled at the pre-metamorphic stage. Eyes were then fixed in RNAlater (Thermo Fisher Scientific, Waltham, MA, USA) for 24 h at 4 °C and stored at −80 °C until use. Total RNA was extracted from the tissues and purified using the RNeasy Mini Kit (Qiagen) following the manufacturer’s instructions. After verifying the RNA integrity on an Agilent 2100 bioanalyzer (Agilent Technologies, USA), only samples with an RNA integrity number (RIN) equal to or above 8 were used. Libraries were sequenced on a BGISEQ-500 platform and single-end reads of 50 base pairs (bp) length were generated per sample.

### Identification of tissue and stage-specific genes

Good-quality raw reads were aligned against the turbot genome assembly (ASM318616v1) using STAR v2.7.0e alignment software^23^ and subsequently assigned to unique genes with HTseq software v0.10.0^24^. Counts with less than 10 reads in at least 9 of our samples were removed. The read counts matrix was normalized using a median-of-ratios method with the R package DESeq2^25^. We performed pair-wise comparisons of differential gene expression among stages and between tissues belonging to the same stage using DESeq2 and determined as differentially expressed genes (DEG) those with *p*-value < 0.05 and log2fold change > 1. We combined all DEGs, resulting from stages-by-stage comparison, in a single set and clustered in a heatmap to analyze stage-specific genes for any of the individual tissues. ClusterProfiler R package v3.14.3^26^ was used for GO enrichment analysis, using a homemade functional annotation as background.

### Gene expression pattern of tissue-stage interaction

To identify genes related to tissue-time interaction, we used the likelihood ratio test (LRT) statistic with DESeq2 package^25^. We used a full model with tissue, time, and their interaction. We removed tissue:stage interaction term for the reduced model, and identified as significant those genes with *p*-value < 0.05. We clustered the expression pattern of genes identified with LRT using DEGreport^27^ and selected those clusters that correspond to unique expression patterns over time for each tissue. GOs were analyzed using ClusterProfiler for genes that matched expression patterns of interest.

### Ethical statements

Ethical approval (ES360570202002/17/FUN.1/BIOLAN.08/JRM) for all studies was obtained from the Institutional Animal Care and Use Committee of the IIM-CSIC Institute in accordance with the National Advisory Committee for Laboratory Animal Research Guidelines licensed by the Spanish Authority (RD53/2013). This work was in conformance with the European animal directive (2010/63/UE) for the protection of experimental animals.

### Reporting summary

Further information on research design is available in the Nature Portfolio Reporting Summary linked to this article.

## Results

### Morphological changes during turbot metamorphosis: skull bone asymmetry

**Fig. 1:**
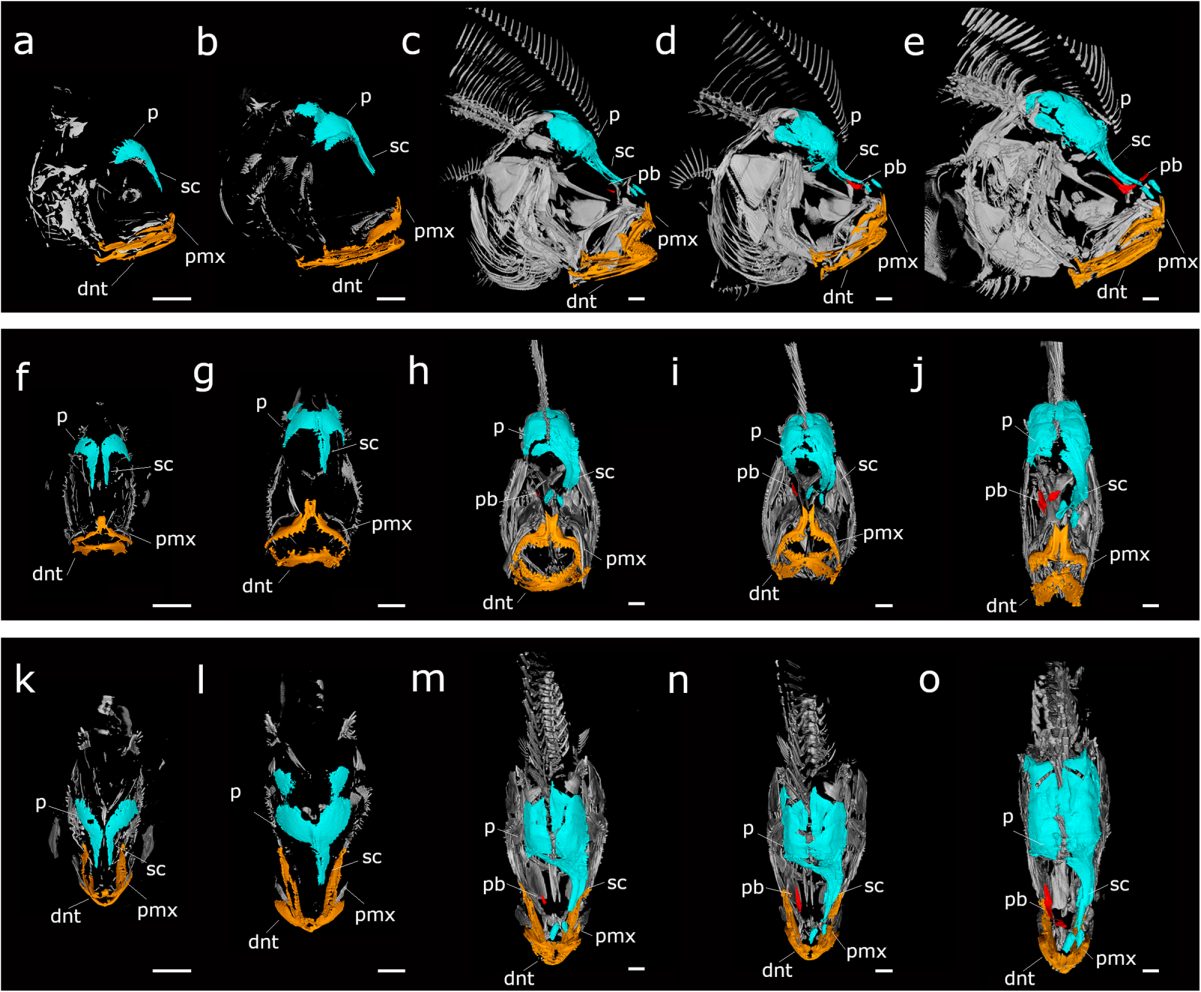
Imaging of the bone development during turbot metamorphosis using light sheet microscopy. Whole animals were visualized with alizarin red staining. For each specimen, a suitable number of tiles were selected to cover the entire sample at lateral position (between 1 × 1 and 4 × 4). The images were captured using a 5× NA 0.16 plan-neofluar detection objective and a zoom of 0.5 to achieve a larger field of view. Visualizations of each specimen were created using the Arivis 4D software. **a**–**e** Lateral view, **f**–**j** frontal view and **k**–**o** dorsal view of (**a**, **f**, **k**) 10 dpf, (**b**, **g**, **l**) 15 dpf, (**c**, **h**, **m**) 20 dpf, (**d**, **i**, **n**) 30 dpf and (**e**, **j**, **o**) 57 dpf turbot. Dentary (dnt) (orange color), parietal (p) (blue color), pseudomesial bar (pb) (red color), supraorbital canal (sc) (blue color), pre-maxilla (pmx) (orange color), dpf (days post fertilization). Scale bar, 500 µm.

Alizarin red staining was used to show developmental asymmetry in the skull during the turbot metamorphic process, followed by light sheet multiview imaging fluorescence microscopy system (LSFM) analysis and 3D reconstruction (Fig. 1). We observed that most of the skull bone structures (parietal, parasphenoid, supraorbital canal, and maxillary bones structures, dentary and premaxilla) exist symmetrically in the larval period in the early pre-metamorphic stages (10 dpf) (Fig. 1a, f, k) and the ocular position maintains the axis. The first evidence of asymmetry is observed in late pre-metamorphosis (15dpf) (Fig. 1b, g, l), where the separation between the frontal bones (left parietal and right parietal, blue color) decreases significantly, and the right parietal bone starts to become visibly narrower in anticipation of future ocular migration. However, at this stage, we do not yet observe the twisting of the supraorbital canal bone and the presence of nasal bone, which is drastically accentuated in early metamorphosis (20 dpf) (Fig. 1c, h, m) following the same direction that the eye migrates until the end of metamorphosis (Fig. 1e). The pseudomesial bar (red color), a peculiar bone present only in flatfishes, appears about the stage at early metamorphosis (20 dpf) (Fig. 1c, h, m) growing dorsally, as observed at the climax of metamorphosis (30 dpf) (Fig. 1d, i, n), and until completed at the end of metamorphosis (57 dpf) (Fig. 1e, j, o).

### Transcriptome assembly and annotation

Eighteen cDNA libraries were created from three different stages of turbot development and from two tissues, the right and left eye and their surrounding tissue, using three independent replicates. More than 20 million 50 bp single-end reads were generated. The raw reads were checked for a good quality score. All clean, high-quality reads were mapped to the turbot reference genome, with averages of 88.96%, 88.73%, 89.19% in the migrant eye and 89.28%, 88.76%, 88.99% in the non-migrant eye at the pre-metamorphic, metamorphic climax, and post-metamorphic stages, respectively.

**Fig. 2:**
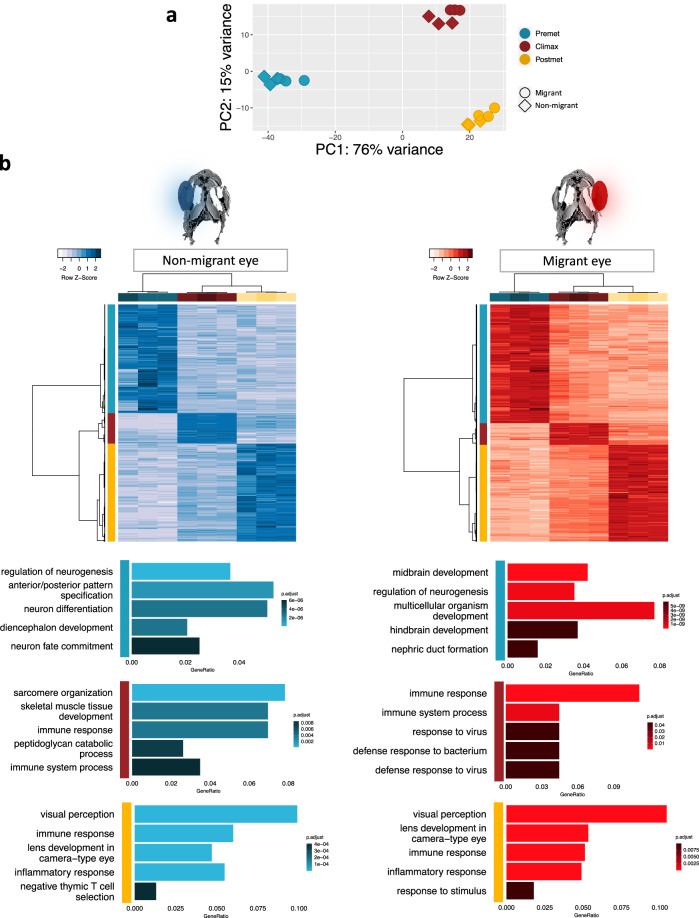
Gene expression profiles in the migrant and non-migrant eye at different stages of development by performing a pairwise comparison between stages. **a** Principal Component Analysis (PCA) plot showing how samples are grouped according to their similarity. Color code: blue, pre-metamorphic stage; red, climax of metamorphosis; yellow, post-metamorphic stage. Shape code: circle, migrant eye; rhombus: non-migrant eye. **b** Heatmap and GOs enrichment of differentially expressed genes clustered only as overexpressed in the pre-metamorphic stage (blue bar), climax (red bar), and post-metamorphic stage (yellow bar) in the non-migrant eye (blue) and the migrant eye (red).

We performed principal component analysis (PCA) and samples belonging to the same stage were clustered together, showing a similar distribution pattern in the migrant and non-migrant eye over time (Fig. 2a).

### Pair-wise comparison using Wald test: stage-specific genes

We investigated the gene expression profile during metamorphosis in turbot eyes, taking three developmental stages as key points. To gain insight into the crucial genes at each stage during the metamorphosis process of turbot, we performed pairwise comparisons among stages in samples belonging to the same tissue. We obtained a total of 2857 differentially expressed genes (DEGs); 1375 genes overexpressed in the pre-metamorphic stage, 308 genes up-regulated during the climax of metamorphosis, and 1174 genes overexpressed in the post-metamorphic stage (Fig. 2b) (Supplementary Data 1–6).

**Fig. 3:**
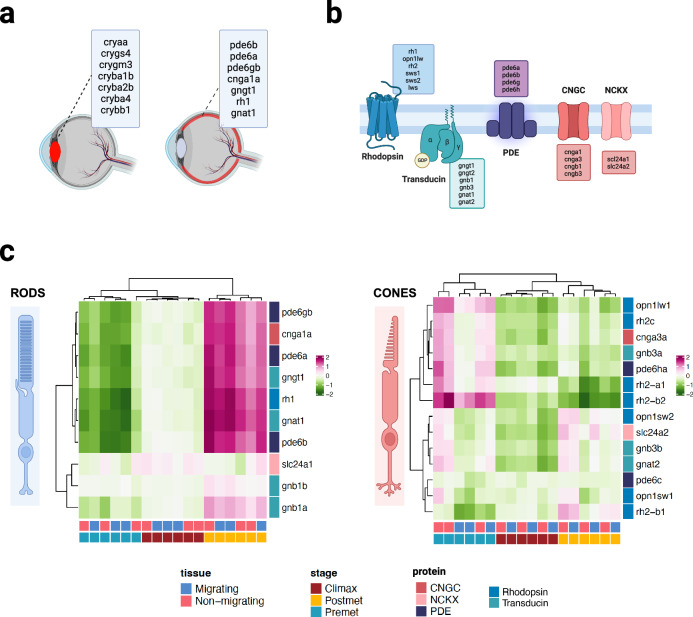
Genes involved in the visual system, phototransduction cascade, and expression profile of these genes in the pre-metamorphic, climax, and post-metamorphic stages. **a** Genes overexpressed at the post-metamorphosis stage in both eyes lens and retinal genes overexpressed at the post-metamorphosis stage in both eyes. **b** Representation of the main proteins involved in the phototransduction cascade in vertebrates. PDE phosphodiesterase, CNGC cyclic nucleotide-gated ion channel, NCKX sodium/calcium-potassium exchanger. In the boxes, the isoforms are expressed during the phototransduction cascade. **c** Gene expression profile throughout pre-metamorphic, climax, and post-metamorphic stages, from migrant and non-migrant eyes samples, of the main genes involved in the turbot phototransduction cascade. The expression profile of isoforms is primarily expressed in rods, and the expression profile of isoforms primarily expressed in cones. Created with BioRender (https://biorender.com).

We performed gene ontology (GO) enrichment analysis with genes overexpressed at each stage in both eyes. Genes upregulated in pre-metamorphic and post-metamorphic stages enriched similar GOs in both eyes (Fig. 2b). Thus, GOs enriched during the pre-metamorphic stage in both the migrant and non-migrant eye were related to early development processes such as regulation of neurogenesis (GO:0050767), anterior/posterior pattern specification (GO:0009952) or cell differentiation (GO:0030154) (Supplementary Data 7 and 8). By performing a GO enrichment analysis on genes that show a peak of expression during the climax of metamorphosis according to Wald test analysis in migrant and non-migrant eyes, we identified eye-specific GO terms (Fig. 2b). Thus, in the non-migrant eye we found enriched biological processes of muscle tissue development such as sarcomere organization (GO:0045214), muscle contraction (GO:0006936), skeletal muscle tissue development (GO:0007519), in contrast to the migrant eye in which we found biological processes related to the immune system such as immune response (GO:0006955) or immune system process (GO:0002376) (Supplementary Data 11 and 12). Interestingly, during the post-metamorphic stage, we also identified significant overlaps of GO terms between eyes that were related to the immune system and ocular development: visual perception (GO:0007601), lens development in the camera-type eye (GO: 0002088), immune response (GO: 0006955) or inflammatory response (GO: 0006954) (Supplementary Data 9 and 10). We highlight unique genes overexpressed in the post-metamorphic stage that is specifically expressed in the lens and photoreceptors outer segment (Fig. 3a, b). Focusing on those genes involved in the phototransduction cascade, we found that their expression pattern is almost identical in both eyes over time (Fig. 3c). However, we discovered differences in expression between isoforms expressed primarily in rods than those expressed in cones.

### Pair-wise comparison using Wald test: tissue-specific genes

**Fig. 4:**
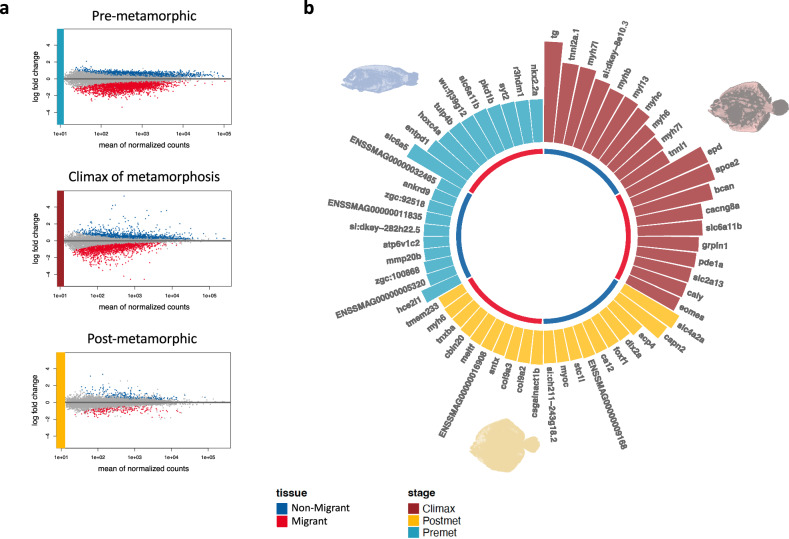
Visualization of differentially expressed genes between the migrant and non-migrant eye in three developmental stages. **a** From top to bottom, gene expression comparison between migrant eye and non-migrant eye in the pre-metamorphic stage, climax of metamorphosis, and post-metamorphic stage, respectively. Blue dots, upregulated genes in the non-migrant eye; red dots, upregulated genes in the migrant eye. **b** Top 10 upregulated genes in each tissue and each stage. Blue semicircular bars represent genes upregulated in the non-migrant eye; red semicircular bars genes upregulated in the migrant eye. The figures illustrating turbot stages from individuals sampled by stage were photographed and edited by the authors.

On the other hand, to know the differences between eyes, we compared the gene expression of the migrant and the non-migrant eye at the pre-metamorphic stage, the climax stage of metamorphosis, and the post-metamorphic stage. We visualized the gene expression dataset using MAplot in order to identify gene expression changes between both eyes in each stage (Fig. 4a). We found large differences during the pre-metamorphic stage and climax, however in post-metamorphic stage, we did not identify marked differences in gene expression. A total of 1890 genes were differentially expressed; 1669 genes were upregulated in migrant eyes as opposed to 221 genes that were upregulated in non-migrant eyes for all the stages. Focusing on each developmental stage, 1026 genes were differentially expressed during pre-metamorphic stage (988 upregulated and 38 down-regulated in the migrant eye) (Supplementary Data 13), 737 genes during the climax of metamorphosis (609 upregulated and 128 genes down-regulated in the migrant eye) (Supplementary Data 14), and 127 genes in the post-metamorphic stage (72 upregulated and 55 genes downregulated in the migrant eye) (Supplementary Data 15). Focusing on the climax stage, we discovered genes expressed in the eye that do not migrate related to fast and slow muscle contraction, such as *tnni2a.1* and *tnni1*, respectively. In addition, we also found different types of myosin, such as *myh7I*, *myl13*, or *myh6*. However, in the migrant eye, we found genes related to neuronal plasticities and synaptic activity, such as *epd*, *bcan*, or *grpin1* (Fig. 4b).

### Tissue-stage expression pattern using LRT

**Fig. 5:**
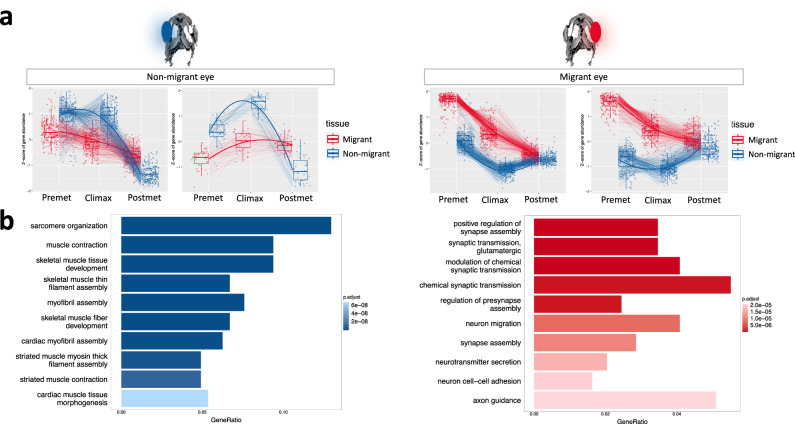
Gene expression pattern and GOs based on LRT analysis. **a** Selected clusters of genes that show a peak of expression during climax, unique to the non-migrant eye (blue), and genes showing a peak of expression during climax, unique to the migrant eye (red). **b** GOs of genes fitting expression pattern shows in (**a**). Blue: GOs of genes belonging to the clusters selected as specific to the non-migrant eye; red: GOs of migrant eye-specific genes.

We used LRT analysis, a statistical tool of the goodness-of-fit between two models, to exhibit genes whose expression varies not only between developmental stages but also between tissues within developmental stages. Using LRT, we identified 2531 genes with adjusted *p*-value < 0.05 (Supplementary Data 16). Genes identified as tissue:developmental stage interaction were clustered, and each of the 2531 genes was assigned to one of the 20 clusters obtained. Those clusters in which expression in one of the tissues showed a concave pattern were selected (Fig. 5a). In the non-migrant eye, we found 289 genes that met these conditions (inter-tissue and developmental stage variability and a concave pattern for the non-migrant eye). Genes specifically expressed during climax in the non-migrant eye enrich GOs such as sarcomere organization (GO:0045214), muscle contraction (GO:0006936), skeletal muscle tissue development (GO:0007519) (Fig. 5b). However, for the migrant eye we did not find such a marked concave pattern. We found a few genes that, during the climax stage of metamorphosis exhibit a single peak. Generally, genes with elevated expression at the climax were also up-regulated in the pre-metamorphic stage. In this case, we found 613 genes that enriched GOs related to the nervous system, such as positive regulation of synapse assembly (GO:0051965), synaptic transmission (GO:0035249), glutamatergic or modulation of chemical synaptic transmission (GO:0050804) (Fig. 5b).

## Discussion

As mentioned earlier, flatfish, including the turbot, undergo one of the most remarkable metamorphoses in the animal kingdom, involving morphological and behavioral changes, transitioning from a symmetric larva with a pelagic lifestyle to a completely asymmetric juvenile with a benthic lifestyle. However, one of the most fascinating aspects of this astonishing metamorphic process is the migration of one eye to the other side of the body.

Our morphological study of this process using advanced imaging techniques such as Light sheet Multiview Imaging fluorescence microscopy (LSFM) system analysis and high-resolution three-dimensional image reconstruction (Fig. 1) reveals that changes in skull shape, particularly in the frontal bones (right and left parietal), take place before ocular migration is readily discernible. These findings support the idea that ocular migration and cranial asymmetry develop as slightly asynchronous processes. In addition, we observed that the pseudomesial bar, a bone unique to flatfishes, forms during the process of eye migration. Therefore, asymmetric presence and asymmetry of the pseudomesial bar on the blind side is possibly responsible for the migration of the right eye during metamorphosis in turbot, similar to what has already been demonstrated in Japanese flounder^28^.

Although the molecular basis seems clear, with thyroid hormone being essential for eye migration in flatfish^29–31^, the network of regulatory mechanisms that allows an eye to move across the head to accommodate its partner remains an undiscovered puzzle where different biochemical processes and physiological functions may be altered.

Therefore, we propose this transcriptomic study in turbot eyes, aiming to connect the genome to gene function by uncovering transcriptionally active genes during the specific right eye migration in turbot that takes place during the metamorphosis process with further adaptations imposed by the ecological environment.

Transcriptomics analyses comparing the two eyes have unexpectedly revealed to us that immune-related genes are significatively up-regulated, particularly in the migrant eye during metamorphosis, compared to other developmental stages analyzed. Because of ocular immune privilege, the mechanism by which the eye limits the local inflammatory response to preserve vision^32^, we would not expect genes such as *dhx58*, *irf7*, or *ifih1* to be overexpressed in eyes. However, our samples include the surrounding ocular tissue, so we cannot confirm that there is no immune privilege in the ocular tissue of metamorphic flatfish. Nevertheless, immune system signaling pathways are activated during tissue reorganization, and inflammation in the eyes promotes axon regeneration after ocular injury^33^. Our data also show an increased expression during the climax and post-metamorphic stage of the *CIITA* gene, which controls *MHCII* expression^34^. *CIITA* expression, probably induced by *IFNγ*^34,35^, could play a role in the maturation and reorganization of the immune system^36^ from larva to juvenile, as well as in recognizing and eliminating larval tissue that drives morphological remodeling around eyes^37^. However, we found no expression differences in the *lc3b* gene, described in autophagy processes around the eye during metamorphosis of *Paralichtlys olivaceus*^38^, between the migrant eye and the non-migrant eye, nor between different stages.

Analyzing eye-specific genes during the climax of metamorphosis, we found specifically in non-migrant eye, overexpressed myosin genes such as *myhb*, *myl13* and *mybpc1*, which play an important role in skeletal muscle development or *klhl41a* involved in sarcomere organization. In contrast, in the migrant eye, we highlight the expression pattern of the *epd* gene, which, interestingly, seems to be involved in the sharpening of synaptic connections in optic nerve regeneration^39^. Although other authors have found no differences in neural projections between the migrant and the non-migrant eye in flatfish^40^, our results show a particular peak of *epd* gene expression during the climax phase of metamorphosis in the migratory eye, and we believe that the peculiar expression pattern of the epd gene shown in our results could open the debate on the asymmetry of visual projections between the eyes in flatfish. On the other hand, GOs related to the nervous system are specifically enriched in the migrant eye. It is clear that adaptative changes in the central nervous system, as well as changes in neuronal connectivity, are necessary for successful settlement^41^. Thus, our data show that these changes are most evident in the migrant eye and/or in its surrounding tissue, identifying asymmetry in nerve tissue^29^.

Accompanying the ocular migration, there is a cranial remodeling that is practically focused on the flexion of the frontal bones and the formation of pseudomesial bar^28^, as shown in our images (Fig. 1). In this study, we identified a specific pattern, with significant overexpression in the migrant-eye side and no significant differences along the timeline in the non-migrating eye, of the *alx3* gene. This gene is specifically expressed in frontonasal neural crest cells and associated with craniofacial disorders^42–44^, and appears to play a key role in craniofacial development in several organisms^45^, exerting control over the specific timing of differentiation and cell morphologies in frontonasal neural crest cells^46^. The expression profile obtained for this gene suggests that localized and differential expression of *alx3* during metamorphosis may contribute to skull restructuring, coordinating the modulation of bone growth that will allow the unique morphological adaptation observed in flatfishes. Although some genes showed asymmetry in expression between eyes, there was no significant differential expression of genes related to the thyroid hormones cascade, such as *trb*, *trab*, and *traa*, between the migrant and non-migrant eye. The expression profile of thyroid hormone action genes in our data is consistent with that described in other studies^47,48^ and is equal in both eyes. This lack of substantial variation is unexpected, given the different developmental pathways of the two eyes. However, the expression of matrix metalloproteinase genes, such as *mmp9*, *mmp14*, or *sparc*, required for tissue resorption and remodeling^49^, is much more pronounced during metamorphosis in the migrating eye compared to the non-migrating eye. In the postmetamorphic stage, the expression of these genes remains elevated in the non-migrating eye.

Based on our findings and previous results from other studies, although one eye undergoes a dramatic migration involving changes described above in muscle, nervous system, and bone, there appear to be no differences in development between the migrant and non-migrant eye. However, we do find significant differences in the development of the eye itself throughout the different stages related to the imposed habitat change. As previously mentioned, the turbot larvae undergo a habitat transition from a pelagic to a benthic state during the metamorphosis process. Life in the benthos is associated with reduced light, which triggers a reorganization of photoreceptors in many fish. This reorganization is critical in establishing the asymmetric pigmentation pattern in the skin of flatfish^50^ and in developing scotopic vision in the retina, as observed in sole^51^. Thus, this new lifestyle requires changes in the expression of various opsins to adapt to the new conditions of light restriction^49,52^. Our results show that rh1, an opsin involved in scotopic vision and expressed in rod photoreceptors, is highly expressed during the post-metamorphic stage when the fish has completed settlement.

In contrast, the gene expression of long-wavelength sensitive opsins and medium-wavelength opsins (*rh2a* and *rh2b*) decrease throughout ontogeny. These changes in the expression pattern of opsins are consistent with the adaptation to the different environments to which flatfish are exposed during development^49^. Additionally, the primary isoforms of the phototransduction cascade present in the postmetamorphic stage are primarily expressed in the rods responsible for scotopic vision. These include *gucy2f, pde6a, pde6g*, and *cnga1a*, which exhibit comparable expression patterns in both eyes. It is important to note that our study also found a decrease in *opn1sw2* gene expression during metamorphosis, which is consistent with previous research. *Opn1sw2* is believed to promote retinoic acid synthesis, which could contribute to inhibiting the metamorphosis process^50^. In addition, we found overexpression of lens genes (*cryaa*, *crygs4*, *crygm3*, *cryba1b*, *cryba2b*, *cryba4*, and *crybb1*), which enrich lens development in camera-type eye GO at the post-metamorphic stage in both eyes. So, our data indicate that the visual function of the migrant eye remains unaltered even after a significant metamorphosis process.

In conclusion, this study uncovers the intricate biological orchestra behind turbot metamorphosis, revealing a fascinating mix of morphological, genetic, and environmental complexities. The study highlights the ocular migration and cranial restructuring, which involves the formation of the pseudomesial bar and frontal bone flexion. These processes are supported by specific genetic expressions, such as the *alx3* gene in craniofacial development and differential activation of immune-related genes. This study intricately links asynchronous eye development in the same individual during turbot metamorphosis with differential expression of musculoskeletal and regenerative genes. The non-migrant eye exhibits specific overexpression of musculoskeletal genes, which are crucial in skeletal muscle development, and this aligns with the relatively static structural demands of this eye. In contrast, the migrant eye exhibits a higher prominence of regeneration genes, specifically the *epd* gene, which is involved in synaptic connections during optic nerve regeneration. This reflects the dynamic and complex developmental processes required for ocular migration. This deeper understanding of the genetic underpinnings of eye-specific development provides novel insights into the intricate coordination of growth and adaptation in response to both internal genetic programming and external environmental challenges.

### Supplementary information

Description of Additional Supplementary Files

Supplementary Data 1

Supplementary Data 2

Supplementary Data 3

Supplementary Data 4

Supplementary Data 5

Supplementary Data 6

Supplementary Data 7

Supplementary Data 8

Supplementary Data 9

Supplementary Data 10

Supplementary Data 11

Supplementary Data 12

Supplementary Data 13

Supplementary Data 14

Supplementary Data 15

Supplementary Data 16

Reporting Summary

## Data Availability

RNA-seq data from all 18 samples representing the three developmental stages have been deposited in the NCBI Gene Expression Omnibus (NCBI GEO). Both raw files in fastq format and processed files can be readily accessed via GEO: Series GSE245741 (https://www.ncbi.nlm.nih.gov/geo/query/acc.cgi?acc=GSE245741)^50^.
