# Supplementary material for: Unraveling the transcriptomic landscape of eye migration and visual adaptations during flatfish metamorphosis
Source: Commun Biol. 2024 Mar 1;7:253. doi: 10.1038/s42003-024-05951-x (PMC10907633; doi:10.1038/s42003-024-05951-x)
Supplement: Supplementary file 1 — Description of Additional Supplementary Files [file 42003_2024_5951_MOESM1_ESM.pdf]

## Description of Additional Supplementary Files

**File name:** Supplementary Data 1
**Description:** Matrix of normalized counts of differentially expressed genes at the pre-metamorphic stage in non-migrant eye.

**File name:** Supplementary Data 2
**Description:** Matrix of normalized counts of differentially expressed genes at the pre-metamorphic stage in migrant eye.

**File name:** Supplementary Data 3
**Description:** Matrix of normalized counts of differentially expressed genes at the climax stage in non-migrant eye.

**File name:** Supplementary Data 4
**Description:** Matrix of normalized counts of differentially expressed genes at the climax stage in migrant eye.

**File name:** Supplementary Data 5
**Description:** Matrix of normalized counts of differentially expressed genes at the post-metamorphic stage in non-migrant eye.

**File name:** Supplementary Data 6
**Description:** Matrix of normalized counts of differentially expressed genes at the post-metamorphic stage in migrant eye.

**File name:** Supplementary Data 7
**Description:** GO enrichment of genes uniquely overexpressed at the pre-metamorphic stage in non-migrant eye.

**File name:** Supplementary Data 8
**Description:** GO enrichment of genes uniquely overexpressed at the pre-metamorphic stage in migrant eye.

**File name:** Supplementary Data 9
**Description:** GO enrichment of genes uniquely overexpressed at the climax stage in non-migrant eye.

**File name:** Supplementary Data 10
**Description:** GO enrichment of genes uniquely overexpressed at the climax stage in migrant eye.

**File name:** Supplementary Data 11
**Description:** GO enrichment of genes uniquely overexpressed at the post-metamorphic stage in non-migrant eye.

**File name:** Supplementary Data 12
**Description:** GO enrichment of genes uniquely overexpressed at the post-metamorphic stage in migrant eye.

**File name:** Supplementary Data 13.
**Description:** List of differentially expressed genes between migrant and non-migrant eye at the pre-metamorphic stage.

**File name:** Supplementary Data 14
**Description:** List of differentially expressed genes between migrant and non-migrant eye at the climax stage.

**File name:** Supplementary Data 15
**Description:** List of differentially expressed genes between migrant and non-migrant eye at the post-metamorphic stage.

**File name:** Supplementary Data 16
**Description:** List of genes obtained from LRT analysis ($p<0.05$).
