# Supplementary material for: Unraveling the transcriptomic landscape of eye migration and visual adaptations during flatfish metamorphosis
Source: Commun Biol. 2024 Mar 1;7:253. doi: 10.1038/s42003-024-05951-x (PMC10907633; doi:10.1038/s42003-024-05951-x)
Supplement: Supplementary file 18 — Reporting Summary [file 42003_2024_5951_MOESM18_ESM.pdf]

nature portfolio

Corresponding author(s): Josep Rotllant

Last updated by author(s): Feb 5, 2024

Reporting Summary

Nature Portfolio wishes to improve the reproducibility of the work that we publish. This form provides structure for consistency and transparency in reporting. For further information on Nature Portfolio policies, see our Editorial Policies and the Editorial Policy Checklist.

Statistics

For all statistical analyses, confirm that the following items are present in the figure legend, table legend, main text, or Methods section.

n/a	Confirmed	
☐	☒	The exact sample size (*n*) for each experimental group/condition, given as a discrete number and unit of measurement
☐	☒	A statement on whether measurements were taken from distinct samples or whether the same sample was measured repeatedly
☐	☒	The statistical test(s) used AND whether they are one- or two-sided<br>*Only common tests should be described solely by name; describe more complex techniques in the Methods section.*
☒	☐	A description of all covariates tested
☐	☒	A description of any assumptions or corrections, such as tests of normality and adjustment for multiple comparisons
☒	☐	A full description of the statistical parameters including central tendency (e.g. means) or other basic estimates (e.g. regression coefficient) AND variation (e.g. standard deviation) or associated estimates of uncertainty (e.g. confidence intervals)
☒	☐	For null hypothesis testing, the test statistic (e.g. *F*, *t*, *r*) with confidence intervals, effect sizes, degrees of freedom and *P* value noted<br>*Give P values as exact values whenever suitable.*
☒	☐	For Bayesian analysis, information on the choice of priors and Markov chain Monte Carlo settings
☐	☒	For hierarchical and complex designs, identification of the appropriate level for tests and full reporting of outcomes
☒	☐	Estimates of effect sizes (e.g. Cohen's *d*, Pearson's *r*), indicating how they were calculated

Our web collection on statistics for biologists contains articles on many of the points above.

Software and code

Policy information about availability of computer code

Data collection	No data collection software was used as it was provided by a third party.
Data analysis	STAR v2.7.0e for align reads and subsequently assigned to unique genes with HTseq software v0.10.0.<br>The read counts matrix was normalized using a median-of-ratios method with the R package DESeq2 v1.42.0.<br>We performed pair-wise comparisons of differential gene expression among stages and between tissues belonging to the same stage using DESeq2, and determined as differentially expressed genes (DEG) those with p-value < 0.05 and log2fold change > 1. We combined all DEGs, resulting from stages-by-stage comparison, in a single set and clustered in a heatmap to analyze stage-specific genes for any of the individual tissues. ClusterProfiler R package v3.14.3 was used for GO enrichment analysis.<br>To identify genes related to tissue-time interaction we used the likelihood ratio test (LRT) statistic with DESeq2 package. We clustered the expression pattern of genes identified with LRT using DEGreport v1.38.0.

For manuscripts utilizing custom algorithms or software that are central to the research but not yet described in published literature, software must be made available to editors and reviewers. We strongly encourage code deposition in a community repository (e.g. GitHub). See the Nature Portfolio guidelines for submitting code & software for further information.

Data

Policy information about availability of data

All manuscripts must include a data availability statement. This statement should provide the following information, where applicable:

- Accession codes, unique identifiers, or web links for publicly available datasets
- A description of any restrictions on data availability
- For clinical datasets or third party data, please ensure that the statement adheres to our policy

RNA-seq data from all 18 samples representing the three developmental stages have been deposited in the NCBI Gene Expression Omnibus (NCBI GEO). Both raw files in fastq format and processed files can be readily accessed via GEO Series accession number GSE245741 (https://www.ncbi.nlm.nih.gov/geo/query/acc.cgi?acc=GSE245741).

Research involving human participants, their data, or biological material

Policy information about studies with human participants or human data. See also policy information about sex, gender (identity/presentation), and sexual orientation and race, ethnicity and racism.

Reporting on sex and gender	*Use the terms sex (biological attribute) and gender (shaped by social and cultural circumstances) carefully in order to avoid confusing both terms. Indicate if findings apply to only one sex or gender; describe whether sex and gender were considered in study design; whether sex and/or gender was determined based on self-reporting or assigned and methods used. Provide in the source data disaggregated sex and gender data, where this information has been collected, and if consent has been obtained for sharing of individual-level data; provide overall numbers in this Reporting Summary. Please state if this information has not been collected. Report sex- and gender-based analyses where performed, justify reasons for lack of sex- and gender-based analysis.*
Reporting on race, ethnicity, or other socially relevant groupings	*Please specify the socially constructed or socially relevant categorization variable(s) used in your manuscript and explain why they were used. Please note that such variables should not be used as proxies for other socially constructed/relevant variables (for example, race or ethnicity should not be used as a proxy for socioeconomic status). Provide clear definitions of the relevant terms used, how they were provided (by the participants/respondents, the researchers, or third parties), and the method(s) used to classify people into the different categories (e.g. self-report, census or administrative data, social media data, etc.) Please provide details about how you controlled for confounding variables in your analyses.*
Population characteristics	*Describe the covariate-relevant population characteristics of the human research participants (e.g. age, genotypic information, past and current diagnosis and treatment categories). If you filled out the behavioural & social sciences study design questions and have nothing to add here, write "See above."*
Recruitment	*Describe how participants were recruited. Outline any potential self-selection bias or other biases that may be present and how these are likely to impact results.*
Ethics oversight	*Identify the organization(s) that approved the study protocol.*

Note that full information on the approval of the study protocol must also be provided in the manuscript.

Field-specific reporting

Please select the one below that is the best fit for your research. If you are not sure, read the appropriate sections before making your selection.

☐ Life sciences ☐ Behavioural & social sciences ☒ Ecological, evolutionary & environmental sciences

For a reference copy of the document with all sections, see nature.com/documents/nr-reporting-summary-flat.pdf

Ecological, evolutionary & environmental sciences study design

All studies must disclose on these points even when the disclosure is negative.

Study description	We show a global view through the creation of a general map of gene expression during the metamorphic process, encompassing both migrant and non-migrant eyes during three different key stages in turbot (3 replicates/stage). Our research uncovers significant differences between migrant and non-migrant eyes of turbot in the pre-metamorphic and climax phases, emphasizing genetic disparities crucial for benthic adaptation.
Research sample	Three key stages in turbot (Scophthalmus maximus) development were defined based on Al-Maghazachi and Gilson and Suarez-Bregua's morphological criteria. Fish reared under standard production conditions at 18ºC, were collected from three metamorphic stages: pre-metamorphic (15 dpf), symmetrical larvae; climax of metamorphosis (30 dpf), migratory eye is visible from the other side of the body; post-metamorphic stage (57 dpf), both eyes lodged in the ocular side. Three independent replicates per stage were collected and fish were euthanized with a lethal dose of MS-222 (500 mg/L for 30-40 min) (Sigma-Aldrich, USA).

Sampling strategy	We have a total of 18 samples divided into 3 developmental stages and two tissues. For each sample we have three biological replicates, which gives us sufficient representation of the stage of study when using next generation sequencing techniques.
Data collection	Eyes were fixed in RNAlater (Thermo Fisher Scientific, Waltham, MA, USA) for 24h at 4ºC and stored at -80ºC until use. Total RNA was extracted from all tissues (n=5, per stage) and purified using the RNeasy Mini Kit (Qiagen) following the manufacturer's instructions. After verifying the RNA integrity on an Agilent 2100 bioanalyzer (Agilent Technologies, USA), only samples with an RNA integrity number (RIN) equal to, or above 8 were used. RNA extractions were performed by lab members. Libraries were sequenced on a BGISEQ-500 platform and single-end reads of 50 base pairs (bp) length were generated per sample by BGI.
Timing and spatial scale	*Indicate the start and stop dates of data collection, noting the frequency and periodicity of sampling and providing a rationale for these choices. If there is a gap between collection periods, state the dates for each sample cohort. Specify the spatial scale from which the data are taken*
Data exclusions	No data were excluded
Reproducibility	Once the RNA was extracted from each sample and sequenced, the replicates were checked by PCA, heatmaps, library size, etc. to ensure that they were valid replicates and therefore checked for reproducibility.
Randomization	The different groups were selected according to morphological studies previously described in other studies. We selected three different stages and two different tissues.
Blinding	Blinding was not relevant to our study. Our results are not conditioned by human perception. They are accurate sequencing data.

Did the study involve field work? ☐ Yes ☒ No

Reporting for specific materials, systems and methods

We require information from authors about some types of materials, experimental systems and methods used in many studies. Here, indicate whether each material, system or method listed is relevant to your study. If you are not sure if a list item applies to your research, read the appropriate section before selecting a response.

Materials & experimental systems

n/a	Involved in the study
☒	☐ Antibodies
☒	☐ Eukaryotic cell lines
☒	☐ Palaeontology and archaeology
☐	☒ Animals and other organisms
☒	☐ Clinical data
☒	☐ Dual use research of concern
☒	☐ Plants

Methods

n/a	Involved in the study
☒	☐ ChIP-seq
☒	☐ Flow cytometry
☒	☐ MRI-based neuroimaging

Animals and other research organisms

Policy information about studies involving animals; ARRIVE guidelines recommended for reporting animal research, and Sex and Gender in Research

Laboratory animals	Scophthalmus maximus: 15 days post fertilization, 30 dpf and 57 dpf. Supplied by Grupo Nueva Pescanova.
Wild animals	*Provide details on animals observed in or captured in the field; report species and age where possible. Describe how animals were caught and transported and what happened to captive animals after the study (if killed, explain why and describe method; if released, say where and when) OR state that the study did not involve wild animals.*
Reporting on sex	Our analyses are not based on sex since the age of the animals is prior to sexual maturation. The interest of the study is temporally prior to sexual maturation.
Field-collected samples	The study did not involve samples collected from the field.
Ethics oversight	Ethical approval (ES360570202002/17/FUN.1/BIOLAN.08/JRM) for all studies was obtained from the Institutional Animal Care and Use Committee of the IIM-CSIC Institute in accordance with the National Advisory Committee for Laboratory Animal Research Guidelines licensed by the Spanish Authority (RD53/2013). This work was in conformance with the European animal directive (2010/63/UE) for the protection of experimental animals.

Note that full information on the approval of the study protocol must also be provided in the manuscript.

Plants

Seed stocks	*Report on the source of all seed stocks or other plant material used. If applicable, state the seed stock centre and catalogue number. If plant specimens were collected from the field, describe the collection location, date and sampling procedures.*
Novel plant genotypes	*Describe the methods by which all novel plant genotypes were produced. This includes those generated by transgenic approaches, gene editing, chemical/radiation-based mutagenesis and hybridization. For transgenic lines, describe the transformation method, the number of independent lines analyzed and the generation upon which experiments were performed. For gene-edited lines, describe the editor used, the endogenous sequence targeted for editing, the targeting guide RNA sequence (if applicable) and how the editor was applied.*
Authentication	*Describe any authentication procedures for each seed stock used or novel genotype generated. Describe any experiments used to assess the effect of a mutation and, where applicable, how potential secondary effects (e.g. second site T-DNA insertions, mosiacism, off-target gene editing) were examined.*
